# Prolonging Survival of Corneal Transplantation by Selective Sphingosine-1-Phosphate Receptor 1 Agonist

**DOI:** 10.1371/journal.pone.0105693

**Published:** 2014-09-12

**Authors:** Min Gao, Yong Liu, Yang Xiao, Gencheng Han, Liang Jia, Liqiang Wang, Tian Lei, Yifei Huang

**Affiliations:** 1 Department of Ophthalmology, Chinese PLA General Hospital. Beijing, China; 2 Department of Ophthalmology, Beijing Chaoyang Hospital, Capital Medical University, Beijing, China; 3 Department of Ophthalmology, Chinese PLA Air Force General Hospital, Beijing, China; 4 Laboratory of Immunology, Institute of Basic Medical Sciences. Beijing, China; Temple University School of Medicine, United States of America

## Abstract

Corneal transplantation is the most used therapy for eye disorders. Although the cornea is somewhat an immune privileged organ, immune rejection is still the major problem that reduces the success rate. Therefore, effective chemical drugs that regulate immunoreactions are needed to improve the outcome of corneal transplantations. Here, a sphingosine-1-phosphate receptor 1 (S1P1) selective agonist was systematically evaluated in mouse allogeneic corneal transplantation and compared with the commonly used immunosuppressive agents. Compared with CsA and the non-selective sphingosine 1-phosphate (S1P) receptor agonist FTY720, the S1P1 selective agonist can prolong the survival corneal transplantation for more than 30 days with a low immune response. More importantly, the optimal dose of the S1P1 selective agonist was much less than non-selective S1P receptor agonist FTY720, which would reduce the dose-dependent toxicity in drug application. Then we analyzed the mechanisms of the selected S1P1 selective agonist on the immunosuppression. The results shown that the S1P1 selective agonist could regulate the distribution of the immune cells with less CD4+ T cells and enhanced Treg cells in the allograft, moreover the expression of anti-inflammatory cytokines TGF-β1 and IL-10 unregulated which can reduce the immunoreactions. These findings suggest that S1P1 selective agonist may be a more appropriate immunosuppressive compound to effectively prolong mouse allogeneic corneal grafts survival.

## Introduction

Corneal transplantation is the most common therapy for many corneal diseases [1]. Compared with many other organs, the cornea is relatively an immune-privileged organ for the absence of immunologic tissues [2]. However, the success rate of corneal transplantation is less than what is generally expected due to the existence of major histocompatibility complex class I and II molecules and indigenous professional antigen-presenting macrophages in the corneal [3]–[5]. Several pathways have been revealed through which corneal allograft would be immune rejected. It has been demonstrated that recognition of donor MHC by recipient CD4 + T-cell was confirmed to play a central role in the corneal allograft rejection [6]. Additionally, some researchers have revealed that CD4 + CD25 + forkhead box P3 (Foxp3) + T regulatory (Treg) cells are important for the survival of corneal allograft [7]. In the past years, various immunomodulatory strategies have been used in experimental corneal transplantation, such as anti-T-cell receptor and T cell depletion, manipulation of co-stimulatory molecule function [8], including Cyclosporine A (CsA) and FTY720. With the application of these drugs in corneal transplantations, beneficial effects have been acquired to some extent. However, the overall efficacies of current used drugs are not yet satisfied and more effective immunoregulatory drugs of low side effect need to be developed for improving the outcome of corneal transplantations in clinic.

Cyclosporine A (CsA), a typical corticosteroids which can causes a significant reduction in interleukin 2-induced corneal neovascularization, is one of the most general used immunosuppression in the patients for prevention of rejection in corneal transplantation [9] It binds to an intracellular protein called cyclophilin and inactivates calcineurin to inhibit IL-2 and lymphocyte production, limiting the activity of CD4+ and CD8+ lymphocytes. However, for high-risk patients, especially in long term treatment, CsA does not reduce the incidence of rejection nor improve the rate of graft clarity [10], [11].

FTY720, a synthetic structural analog of myriocin, was also frequently used as an immune-modulator to inhibit rejection after corneal transplantation in animal models. Different from CsA, FTY720 does not inhibit T cell activation and proliferation, it exerted an effect on certain lymphocyte populations in the immunosuppression process [12], [13]. FTY720 was used in many organ transplants, such as the kidney and liver transplantation, and demonstrated substantial efficacy in prolonging survival of the grafts through regulating the S1P1 [13], [14]. It has been confirmed that S1P1 was essential for lymphocyte recirculation and regulated egress of lymphocytes from the thymus and peripheral lymphoid organ. However, FTY720 can cause the drawback of bradycardia as well as other side effects. These effects may be caused by its non-specificity, because FTY720 acts as a full agonist on four known sphingosine-1-phosphate (S1P) receptors (S1P1, S1P 3–5) [15]. We supposed that a S1P1 selective agonist may be a more effective drug for prevention of rejection in corneal transplantation and avoid the disadvantages of FTY720 due to the non-specificity. Thus in the study, we employed a S1P1 selective agonist and systematically compared its efficacy with the commonly used immunoregulatory drugs in mouse allogeneic corneal transplantation.

## Materials and Methods

### Chemical compounds

**Figure 1:**
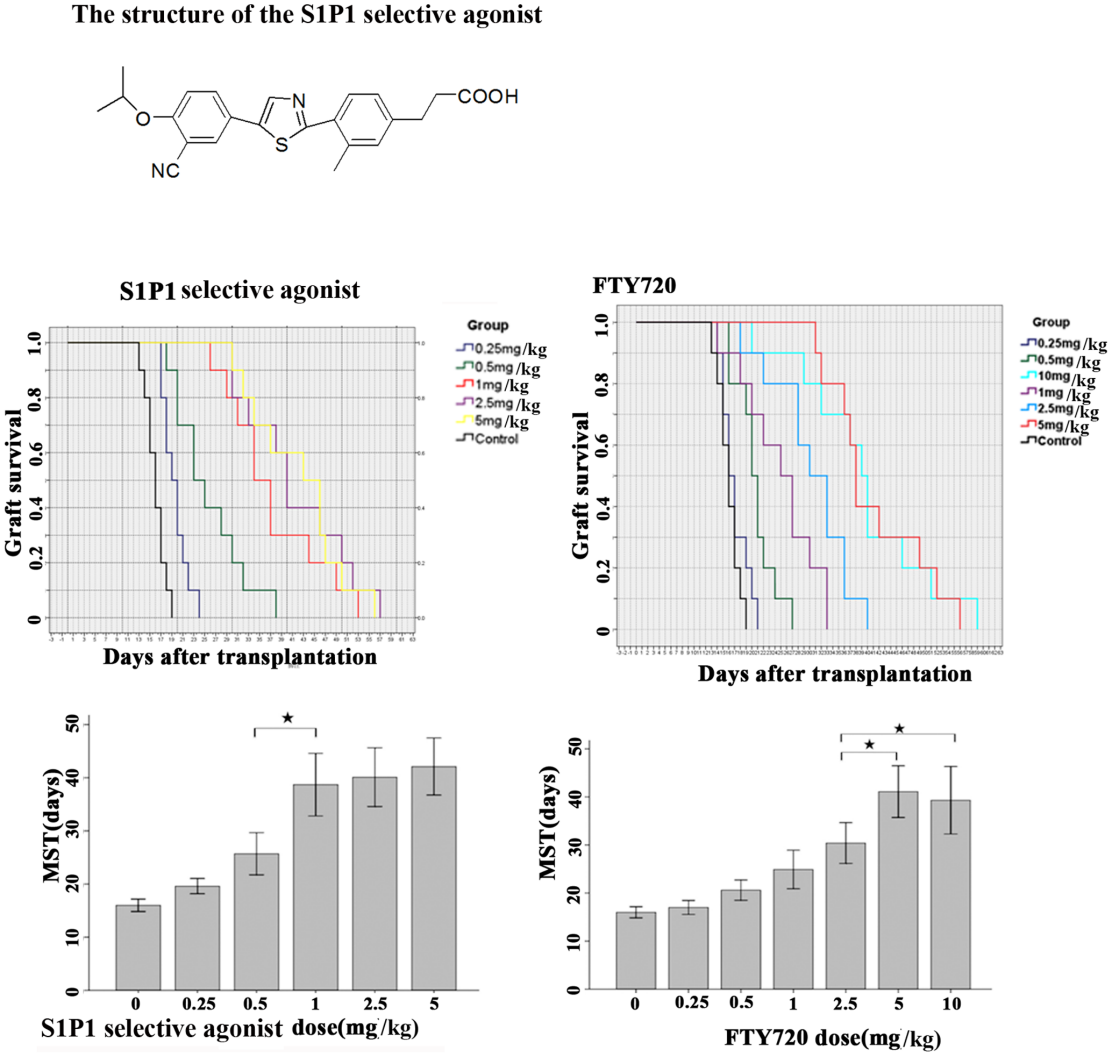
The structure of S1P1 selective agonist used in the study, as well as the dose optimization of S1P1 selective agonist and non-selective agonist FTY720. The dose optimization of S1P1 selective agonist and non-selective FTY720.BALB/c mice received allogeneic corneal transplantation and then were treated with selective S1P1 agonist or FTY720 of different dose(S1P1 selective agonist: 0.25 mg, 0.5 mg, 1 mg, 2.5 mg, 5 mg/kg/d; FTY720: 0.25 mg, 0.5 mg, 1 mg, 2.5 mg, 5 mg, 10 mg/kg/d; saline was used as 0 dose; n = 10 for each group). The optimal dose was determined based on the survival curve and median survival time (MST). The results indicated that the optimal doses for S1P1 selective agonist and FTY720 in mouse allogeneic corneal transplantation were 1 mg/kg/d and 5 mg kg/d.

FTY720 and Cyclosporin A were purchased from Sigma (SML0700 and 30024). The selective S1P1 agonist used in the study was synthesized by Beijing Institute of Pharmacology & Toxicology, Academy of military medical sciences. This compound was designed and synthesized as the non-phosphate S1P1 receptor agonists. The structure of this compound was shown below. EC50 of these selective S1P1 agonists was 0.18 nM, and the affinity to S1P1 was 55000 folds higher than to S1P3, 10000 folds higher than to S1P2 and S1P4, 600 folds higher than to S1P5. The details about the compound could be found in the previous report [16]. The structure of the S1P1 selective agonist used in the study was shown in Figure 1.

### Experiment animals

Male inbred C57BL/6 and BABL/c mice used for orthotopic corneal transplantation were purchased from the Experimental Animal Center of Academy of Military Medical Sciences, Beijing, China. These two inbred mouse strains are fully mismatched for major histocompatibility complex and multiple minor histocompatibility antigens. All animals were treated in accordance with the ARVO statement for the Use of Animal in Ophthalmic and Vision Research. This work has been reviewed and approved by the Institutional animal care and use committee, institute of laboratory animal science, Chinese PLA General Hospital. The performance site for these animal works is an AAALAC accredited institution. All of the donor and recipient mice were anesthetized by intraperitoneal injection of 3% pentobarbital sodium before any surgical procedures. All the sacrificed animals were treated by CO2 asphyxia death. The human endpoints were used when the immunological rejection of corneal transplantation was observed in with severe opacity (four grade), edema (four grade) and neovascularization (four grade).

### Orthotopic allogeneic corneal transplantation

A total of 216 BALB/c mice that received corneal grafts from C57BL/6 donors were used in the study [17]. Firstly, 120 BALB/c mice were used to determine the optimal dose of selective S1P1 and non-selective FTY720. They received allogeneic corneal transplantation(details in bellow) and then were treated with selective S1P1 agonist or FTY720 of different dose(selective S1P1 agonist: 0.25 mg, 0.5 mg, 1 mg, 2.5 mg, 5 mg/Kg/d; FTY720: 0.25 mg, 0.5 mg, 1 mg, 2.5 mg, 5 mg, 10 mg/Kg/d; saline was used as 0 dose; n = 10 for each group). The optimal dose was determined based on the survival curve and median survival time (MST).

Then, 96 mice were performed corneal transplantation and randomly divided into four groups: control group, cyclosporine A (CsA) treated group; FTY720 treated group and selective S1P1 agonist treated group (n = 24 for each group). The surgery was performed as previously described [18], [19]. Briefly, before surgical procedures, the donor and recipient mice were anesthetized by intraperitoneal injection of 3% pentobarbital sodium (80 mg/Kg body weight; Nembutal; Beijing Chemical Co., Beijing, China). A tropicamide-phenylephrine ophthalmic solution (Santen Pharmaceutical Co., Ltd, Japan) was topically applied to dilate the pupil of both the donors and recipients. The corneal button was placed into a balance salt solution (BSS TM; Alcon, Fort Worth, TX) before grafting for less than half an hour. The donor corneal graft was sutured into a 1.5 mm BABL/c recipient corneal bed with 8–10 interrupted 11–0 nylon sutures undermicroscope. The anterior chamber of eye was restored with disinfected air at the end of surgery. After surgery, if the abnormal events occurred, including cataract, corneal graft rupture, perforation, anterior synechia and hyphema, the animals should be removed and new animals receiving the surgery were supplemented. Corneal sutures were removed 10 days after surgery. Control group mice received intraperitoneal injection of saline without any drugs. Other three groups mice received cyclosporine A (5 mg/kg/d), FTY720 (optimal) and S1P1 selective agonist (optimal), respectively. The drugs in all groups were injected daily for 14 days. The observation period lasted until the last corneal was immune rejected.

### Clinical evaluation and examination after orthotopic corneal transplantations

After transplantation, the corneal opacity as well as neovascularization was evaluated by microscopy daily until day 30. Donor corneal opacity score (0–4), edema score (0–2), and neovascularization score (0–4) were graded according to preciously described criteria [20]. The animals with complications after transplantation surgery such as intraocular hemorrhage, infection or cataract, were excluded.

### Histological and Immunohistological Evaluation

Fourteen days after the grafting procedure, 4 eyes receiving grafts in each group were enucleated and fixed in 10% formaldehyde solution. The samples were embedded in paraffin for preparation of 3 microns paraffin-embodied sectioned. Then, slides with sections were deparaffinized performed with hematoxylin and eosin (H&E) staining for histological examination. For immunohistological analysis, the slides were also deparaffinized and then processed for immunohistochemical staining with anti-CD4(Sigma) and anti-vWF(Sigma) according to the previous reported [17] antibodies. Briefly, after deparaffinization, the slides were rehydrated and heat-induced antigen retrieval was performed. After blocking with 10% normal horse serum for 15 min, the sections were immunostainned using the primary antibodies and incubated overnight at 4°C. The redundant antibodies were washed with Phosphate Buffered Saline. The corresponding secondary antibodies were added and incubated for 2 hours at room temperature.

### Flow cytometric analysis

The T cell phenotype in cervical lymph nodes, mesentery lymph node, spleens and peripheral blood of six mice in each group was analyzed by flow cytometer at day 14 after transplantation. Treg cells were detected by a Mouse Regulatory T cell Staining Kit (APC labeled Foxp3 FJK-16s, FITC-labeled CD4, and PE-labeled CD25) from eBioscience (San Diego, CA, USA). Data were acquired using FACS Calibur flow cytometer (BD Biosciences, Phamingen, CA, USA), and analyzed using Winmid 2.9 software (Scripps Institute, La Jolla, CA). The cells were gated for CD4 + and the percent of CD4+CD25+FoxP3 + cells were calculated. The results are given as the percentage of CD4+T and CD4 CD25 FoxP3 + T cells.

### Real time PCR

Total cellular RNA of corneal button (3 mm diameter in the central cornea) was extracted using the TRIzol reagent (Invitrogen, Carlsbad, CA) and liquid nitrogen according to routine procedure. Gene expression was examined in an iCycleriQ Real-time PCR Detection System (Bio-Rad, Hercules, CA) using the SYBR Green Real-time PCR Master Mix (TOYOBO, Osaka, Japan) with respective real-time quantitative PCR primers [17].

### ELISA

Standard ELISA assays were used to measure the serum autoantibody production on day 14 after transplantation. To prepare the serum samples, peripheral bloods samples from mice were centrifuged at 1,000 rpm for 20 min at 4°C, and the supernatant immediately separated from the pellet. Serum levels of IL-2, IL-10, TGF-b1, and IFN-r were measured using the corresponding ELISA kit according to the manufacture's guideline (MultiSciences Biotech Co., Ltd, Hangzhou, China).

### Statistical Analysis

Actuarial graft survival was analyzed using the Kaplan-Meier survival method, and the log-rank test was used to examine statistical differences among the groups. A One-way ANOVA with least significant difference (LSD) test was used in comparison between the four groups. A p value <0.05 was considered significant.

## Results

### The optimal doses of S1P1 selective agonist and non-selective FTY720

As shown in Fig 1, both S1P1 selective agonist and FTY720 promoted the graft survival in a dose-dependent manner. For S1P1 selective agonist, no significant improvement in graft survival was observed when the dose was more than 1 mg/kg/d, while for FTY720, the graft survival increased with the drug dose until the dose was up to 5 mg/kg/d. The results indicated that the optimal doses for S1P1 selective agonist and FTY720 in mouse allogeneic corneal transplantation were 1 mg/kg/d and 5 mg/kg/d. respectively. This was consistent with our presumption that S1P1 selective agonist would be more effective than non-selective FTY720. In the following experiment, the optimal doses were selected.

### S1P1 selective agonist prolongs the survival time of corneal grafts

**Figure 2:**
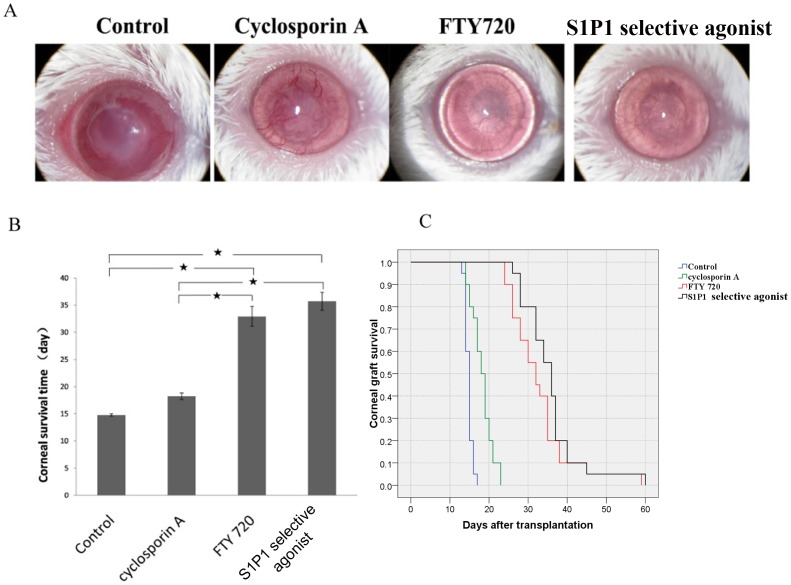
The effects of sphingosine-1-phosphate receptor agonist on the survival of corneal allografts. **A.** Opacity, edema and neovascularization of corneal grafts after treatment by S1P1 selective agonist, FTY720, Cyclosporin A and control. **B.** Mean survival time of rejection free graft after treatment by S1P1 selective agonist, FTY720, Cyclosporine A and control. **C.** Survival curves of rejection free graft after treatment by S1P1 selective agonist, FTY720, Cyclosporin A and control. Green curve: Cyclosporin A (5 mg/kg/d) treated recipients. Red curve: FTY720 (5 mg/kg/d) treated recipients. Black curve: S1P1 selective agonist (1 mg/kg/d) treated recipients. Twenty mice were used for each group.

To investigate the effectiveness of S1P1 selective agonist on suppressing the immune rejection after corneal transplantation and compare its efficacy with other commonly used immunosuppressive agents. Animals receiving a corneal graft were treated with intraperitoneal injection of S1P1 selective agonist, FTY720, CsA and saline, respectively. 14 days post transplantation, obvious immunorejection for corneal transplantation was observed in the control group with severe opacity (four grade), edema (four grade) and neovascularization (four grade), while in comparison, the immune rejection in the CsA treated group was significantly attenuated that the opacity (three grade), edema (one grade) and neovascularization (three grade) was improved. The recipients treated by FTY720 and S1P1 selective agonist were not observed with obvious immune response that they shew clear corneal stromal and a golden circle in the anterior chamber until 30 days post transplantation (Figure 2A). After corneal transplantation, the survival time of grafts in each animal was followed up and the mean survival time (MST) of grafts in each group was counted. Compared with the control group, FTY720 and S1P1 selective agonist treatment significantly prolonged the mean survival time of grafts for more than 15 days. Although CsA treatment somewhat improved the MST of grafts, no statistically significance was observed compared with the control (Figure 2B). To show the dynamic changes of the graft survival rates in different groups, the survival curve was mapped (Figure 2C) which provided a direct view for the efficacies of different drugs, that FTY720 and S1P1 selective agonist worked better than CsA for the survival of corneal grafts.

### Regulation of host inflammatory response

**Figure 3:**
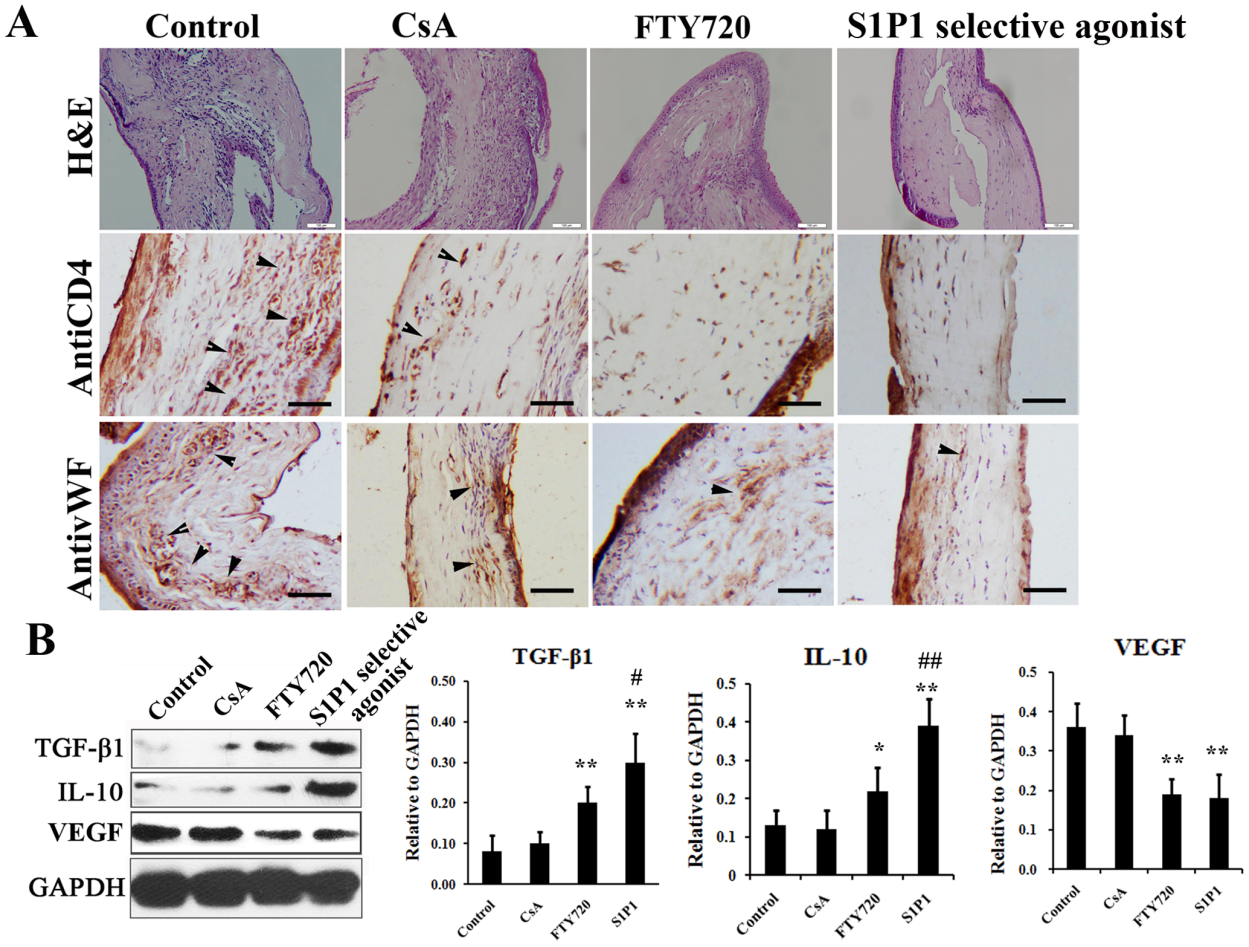
Inflammatory reaction and kidney injury of the recipients after treatment by sphingosine-1-phosphate receptor agonist. **A.** A Histopathology of the corneal grafts stained with hematoxylin-eosin, CD4 antibody and vWF antibody at day 14 after treatment by S1P1 selective agonist, FTY720, Cyclosporine A and control(Arrows indicated positively stained cells, H&E staining Bar  = 100 µm; immunostaining Bar  = 25 µm). **B.** The proteins levels of IL-10, TGF-βand VEGF were detected by western blot at day 14 after treatment by S1P1 selective agonist, FTY720, Cyclosporine A and control. *P<0.05 compared with control and CsA groups; **P<0.01 compared with control and CsA groups; #P<0.05 compared with control and FTY720 group; ##P<0.05 compared with control and FTY720 group.

To further understand the immunosuppressive efficacy of the S1P1 selective agonist for the corneal transplantation, we analyzed the distribution of the immune cells using non-specific H&E staining and specific immunostaining staining against CD4. By H&E staining, it could be observed that many cells aggregated(many nuclei were observed) in the grafts from control group and CsA group, indicating that apparent immune reaction was provoked, while the aggregated cells were significant less both in FTY720 and S1P1 selective agonist groups (Figure 3A). The results from H&E staining were further confirmed by immunohistochemical staining. The immunostaining against CD4 showed that obvious CD4+ cells infiltrated the allograft in control and topical CsA treated samples, while in contrast, no significant infiltration of CD4+ cells were observed in the FTY720 and S1P1 selective agonist groups, especially the S1P1 selective agonist group. To evaluate the neovascularization in allograft, the immunostaining against vWF was performed and a consistent result was obtained. As shown in Fig 3A, many vascular structures could be observed in control and CsA group, which were much less in FTY720 and S1P1 selective agonist groups.

In addition to histological and immunohistochemical staining, Western blotting analysis of host inflammatory response was also performed. As shown in Fig 3B, we detected the expression of anti-inflammatory cytokines IL-10 and TGF-β1, and found a higher level of these cytokines in S1P1 agonist treated group than that in the other three groups.

To evaluate the neovascularization in allograft, anti-VEGF antibodies were used in Western blotting. The results demonstrated that the expression of VEGF is obviously lower in S1P1 selective agonist treated group than that in control and CsA groups. When compared between S1P1 selective agonist and FTY720 groups, no significant difference was observed in the expression of VEGF (Figure 3B).

### The distribution of the immune cells and the levels of immune-regulation related cytokines

**Figure 4:**
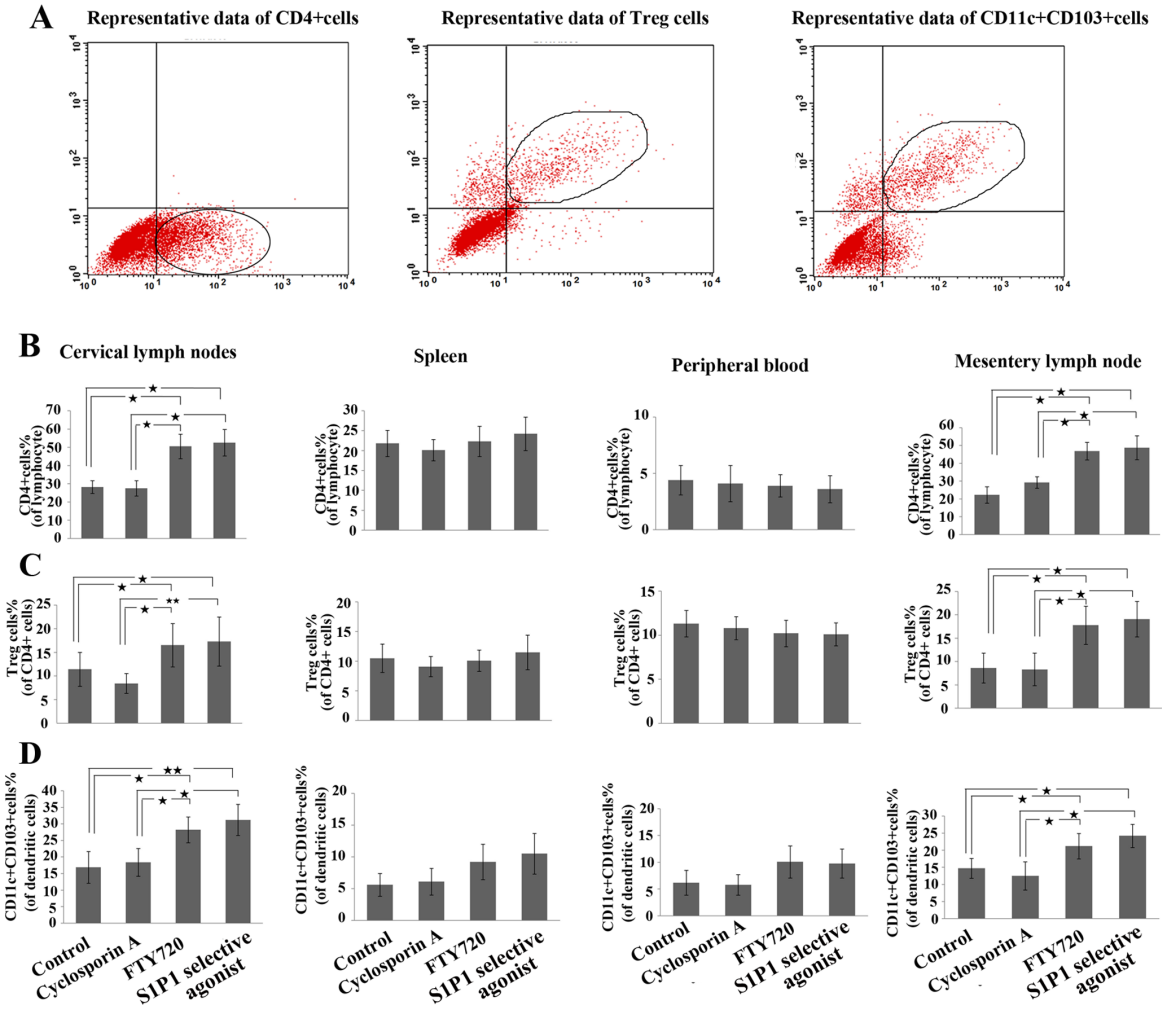
Effects of S1P1 selective agonist to the distribution of the CD4+T cells, CD4+CD25+Foxp3+T(Treg) cellsand CD11c+ CD103+ cells in the lymphoid tissue. (A) Representative flow cytometry of different cell populations; (B)The percentage of the CD4+T cell populationin cervical lymph nodes, spleen, peripheral blood and mesentery lymph node; (V) The percentage ratio Treg cells(to CD4+ cells) in cervical lymph nodes, spleen, peripheral blood and mesentery lymph node;(D) CD11c+ CD103+ cells in cervical lymph nodes (B), spleen (C), peripheral blood (D) and mesentery lymph node. The data was analyzed by flow cytometer at day 14 after treatment by S1P1 selective agonist, FTY720, Cyclosporine A and control. Values represent mean +/− SD, n = 5 mice per group. **p<0.01; *p<0.05.

In our previously research, we found that the FTY720, the non-selective S1P agonist, could suppress immunoreaction by decreasing in the number of CD4 + T cells in the corneal. Moreover, this was also verified by Lynch Kevin's studies [21]. So we hypothesized that the S1P1 selective agonist should affect the distribution of lymphocytes. Then we analyzed the distribution of the main immune-regulation related cells: CD4 + positive cells, CD4+CD25+Foxp3+T cells and CD11c+CD103+cells by FACS. We measured these cells in the cervical lymph nodes (CLN), spleen, peripheral blood and mesentery lymph node. The results demonstrated that the mean percentages of CD4+ T cells in cervical and mesenteric lymph nodes of the recipients treated by FTY720 and S1P1 selective agonist were higher than that in the control group, while CD4+CD25+Foxp3+Tcells and CD11c+CD103+cells were significantly less in FTY720 and S1P1 selective agonist groups (Figure 4A and 4D). These measured cells both in the cervical and mesenteric lymph nodes of the CsA treated groups were observed no significant differences compared with that of the control group. Among the four groups, the quantification of CD4+ T cells demonstrated no significant difference in the peripheral blood samples (peripheral blood lymphocytes, PBLs) and the spleens. Similarly, the content of the CD4+CD25+Foxp3+ T cells and CD11c+CD103+ T cells was at a similar level among the four recipient groups (Figure 4B and 4C).

**Figure 5:**
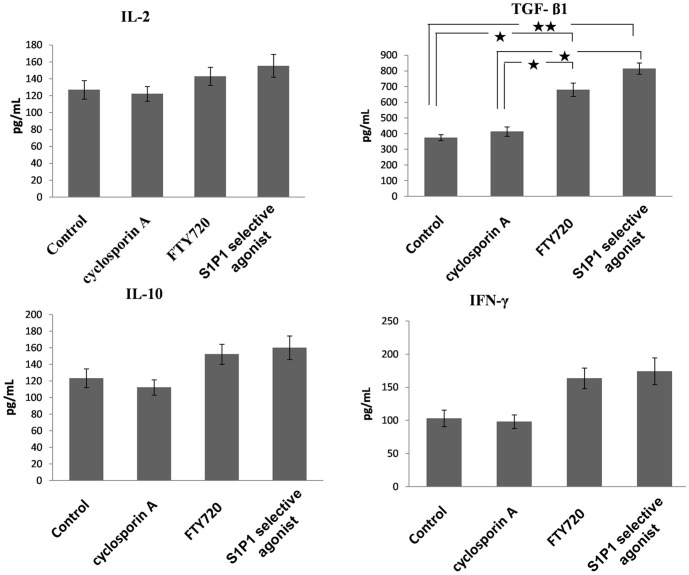
Expression levels of immunoregulation related cytokines in the serum after treatment by the S1P1 selective agonist. The levels of IL-2, TGF-β1, IL-10 and IFN-γ were measured by ELISA at day 14 after treatment by S1P1 selective agonist. Values represent mean +/− SD, n = 5 mice per group. **p<0.01; *p<0.05.

Then, we measured the protein levels of the pro-inflammatory cytokines IL-2, IFN-γ, anti-inflammatory cytokines IL-10 and TGF-b1 in the serum of the four group mice. The levels of TGF-β1 in the S1P1 selective agonist treatment group was higher than that in the control group (p<0.01).The FTY720 treated group also had higher level of TGF-b1 than control group (p<0.05), but not the CsA treated group. However, consistent with previous reports, we did' not find statistically significant difference in the expression of IL-2, IL-10 and IFN-γ after treatment by FTY720 and S1P1 selective agonist. (Figure 5) [22], [23].

### The expression of immune-regulation related genes in the corneal grafts

**Figure 6:**
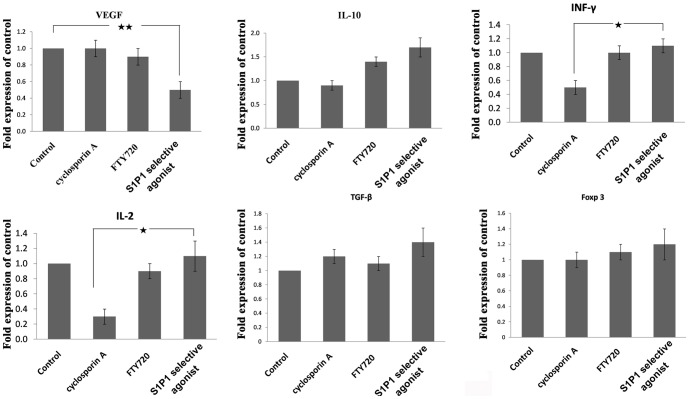
Expression levels of immunoregulation related genes in the in the corneal grafts after treatment by S1P1 selective agonist. mRNA expression levels of *VEGF*, *IL-10*, *TNF-γ*, *IL-2*, TGF-β1 and Foxp3were measured by RT-PCR at day 14 after treatment by S1P1 selective agonist, FTY720, Cyclosporine A and control. Values represent mean +/− SD, n = 5 mice per group. **p<0.01; *p<0.05.

To further investigate the effects of the S1P1 selective agonist, we measured the expression of the major regulate factors by real-time PCR. Compared with the control group, the expression of pro-inflammatory cytokines IL-2 and IFN-r had no significantly changes after treatment by S1P1 selective agonist or FTY720. However, these two pro-inflammatory cytokines were distinctly down regulated after CsA treatment compared with the control group. The corneal expression of TGF-β1 was unregulated by S1P1 selective agonist, which was consistent with the expression pattern of the serum TGF-b1. In addition, we also found that the S1P1 selective agonist treatment, but FTY720, could significantly down regulate the expression of VEGF in the corneal grafts (Figure 6).

## Discussion

Systemic immunosuppression which has beneficial effects in the high risk keratoplasty has been reported [23]. CsA, a drug generally used in clinic, is not sufficient or effective after high risk corneal transplantation [24]. New immunosuppressive agents are needed developing. FTY720 is a new immunosuppressive agent that does not inhibit T cell activation and proliferation, having an effect on certain lymphocyte populations in the immunosuppressant process. However, FTY720, not a specific S1P1 agonist, exists the side effects. In this study, we showed that the selected S1P1 agonist can prolong the corneal graft survival as long as the reported FTY720, which is a non-selected S1P agonist and exist the side effects [25]. In 2011, Robert Wills and his colleagues found a S1P1 selective agonist, and made a series of comparisons with the FTY720. The results shown that the S1P1 selective agonist had excellent pharmacokinetic properties with low oral dose and few side effects on heart rate. In the previous work [26], it has been shown that the cytotoxicity of S1P1 selective agonist (used in present study) was lower than FTY720. Collectively, it may be supposed that the novel agent may possess less side effects in future application than that was reported about FTY720.

In our experiments, we optimized the dose of the S1P1 selective agonist in mice models receiving corneal allografts. Then, we systematically compared the efficacy of the S1P1 selective agonist with the commonly used immunosuppressive drugs CsA and FTY720. We found that the systemic application of S1P1 selective agonist and FTY720 could significantly prolong the survival time of the allografts, while the application of CsA demonstrated no significant difference compared with control group. The recipients treated by S1P1 selective agonist and FYT720 had non-rejected response showing clear corneal stromal and a golden circle in the anterior chamber 30 days post-surgery. These results showed that both non-selective S1P agonist FTY720 and S1P1 selective agonist can both reduce the immunological rejection after corneal transplantation. However, the therapeutic dose of the selective S1P1 agonist was significantly lower than that of FTY720, which may be effective to reduce the potential drug toxicity at high dose. Moreover, the high selectivity of S1P1 agonist would also significantly avoid the potential effects that was due to the non-selective regulation of S1P targets by FTY720, presenting a promising perspective in clinic. The clinic efficacy and safety of the S1P1 selective agonist in organ transplantation deserved further investigation.

Compared to controls, treatment with this S1P agonist or FTY720 was observed to inhibit the growth of vessels on the transplanted corneas. Actually, there have been some evidences showing that the sphingosine-1-phosphate (S1P) could regulate the expression of VEGF and angiogenesis. Christer Betsholtz and his colleagues found that S1P was critical for inhibition of angiogenesis and acquisition of vascular stability. They demonstrated that S1PR1 regulates VEGF-Induced VEGFR2 signaling and internalization. Moreover, S1PR1 signaling could stabilize the junctional of VE-cadherin and inhibit VEGFR2 phosphorylation and downstream signaling. According to these results, it was reasonable the S1P agonist could inhibit the expression of VEGF in the corneal transplation [27].

To further investigate the effect of the S1P1 selective agonist on regulating the immunological rejection, we measured the distribution the immune cells. The flow cytometric analysis showed that there was a statistically significant difference in the percentage of CD4+T cells (both in the cervical and mesenteric lymphnodes) in these mice receiving S1P1 selective agonist or FTY720 treatments compared with those in control group. The results were consistent with the previous studies about FTY720 which was demonstrated showed to inhibited T cells egress from lymphoid organs through modification of S1P receptors. CD4+T cells are the most important T cell population involving in corneal allograft rejection [28] and therefore, we suggest that inhibition of CD4+ T cells in the cornea should be one of the important mechanisms for S1P1 selective agonist and non-selective FTY720 to prolong corneal allograft survival.

In addition, like FTY720 can significantly increase the percentage of Treg cells by reducing the chemotactic response to S1P receptor [29], we found that S1P1 selective agonist significantly enhanced the percentage of CD4+CD25+Foxp3+T cells and CD11c+CD103+ T cells in the cervical lymphoid nodes followed by a change in the CD4+T cell distribution.

TGF-b1 was reported as an anti-inflammatory cytokine. In the study, we found that the serum level of TGF-b1 increased in the S1P1 selective agonist treated mice, indicating that S1P1 agonist may also as a regulator of TGF-b1 [30]. In the analysis of the intra-graft mRNA gene expression of various cytokines, we also found that TGF-β1 and IL-10 mRNA expression in the S1P1 selective agonist group was higher than that in the other groups., This suggests that S1P1 selective agonist may significantly enhance the immune function of Treg cells by increasing levels of TGF-β1 and IL-10 in the corneal. The IL-2 and IFN-r mRNA expressions in the CsA treated group were lower than those in other groups. The result was concordant with the previous studies that reported CsA suppression of T cell proliferation via inhibition of IL-2 and IFN-r synthesis [31]. More interestingly, we found that the expression level of VEGF, which is important for the neovascularization, was obviously down regulated by S1P1 selective agonist [32]. These results suggest that S1P1 selective agonist may be a more appropriate immunosuppressive compound than FTY720.

In summary, our study confirmed that S1P1 selective agonist can effectively prolong the corneal allograft survival in mice as non-selective FTY720 at a significantly lower dose. More importantly, the drug should be safer in application due to its high selectivity in action and markedly reduced dose in administration compared with non-selective FTY720.
